# Preferential binding of 4-hydroxynonenal to lysine residues in specific parasite proteins in plakortin-treated *Plasmodium falciparum*-parasitized red blood cells

**DOI:** 10.1016/j.dib.2015.11.003

**Published:** 2015-11-11

**Authors:** Evelin Schwarzer, Valentina Gallo, Elena Valente, Daniela Ulliers, Orazio Taglialatela-Scafati, Paolo Arese, Oleksii A. Skorokhod

**Affiliations:** a Department of Oncology, University of Torino, Via Santena 5bis, 10126 Torino, Italy; b Department of Pharmacy, University of Napoli “Federico II”, Via D. Montesano 49, 80131 Napoli, Italy

**Keywords:** plakortin, endoperoxide, antimalarial drug, 4-hydroxynonenal, post-translational modifications, Plasmodium falciparum, red blood cell

## Abstract

The data show the frequencies by which the amino acid residues lysine, histidine and cysteine of six proteins of the malaria parasite *Plasmodium falciparum* are post-translationally modified by the lipoperoxydation endproduct 4-hydroxynonenal after challenging the parasitized red blood cell with plakortin. Plakortin is an antimalarial endoperoxide whose molecular anti-parasitic effect is described in Skorokhod et al. (2015) [1]. Plakortin did not elicit hemoglobin leakage from host red blood cells and did not oxidize reduced glutathione.

**Specifications Table**

**Table:** 

Subject area	Biology
More specific subject area	Molecular pharmacology
Type of data	Tables, graphs
How data was acquired	MALDI-TOF spectrometer (MALDI micro MX, Waters, Milford, MA, USA); Luminometer Sirius (Berthold, Pforzheim, Germany)
Data format	Processed data
Experimental factors	Parasitized and non-parasitized red blood cells were kept in in vitro cell culture up to 24 h. Chemical extraction for parameter assessment.
Experimental features	Heme-dependent luminol-enhanced luminescence assay for hemoglobin. Thiol-reaction with DTNB and OD measurement at 412 nm. Mass spectrometric analysis of peptides after trypsin-digestion of proteins.
Data source location	University of Torino, Torino, Italy
Data accessibility	Data are provided with this article

**Value of the data**

• The data show how endoperoxide-elicited oxidative stress might specifically target parasite proteins of functional importance by binding the lipoperoxidation endproduct 4-HNE.
• Data on endoperoxide-elicited modifications of proteins of a unicellular parasite as molecular cause for cell death may be of interest for similar studies with other microorganisms.
• Data for lethal modifications of specific microbial proteins with lipoperoxidation endproducts such as 4-HNE may suggest a novel strategy for high throughput drug research, e.g. antimalarials.
• The data on host red blood cells (RBC) intactness may be useful to monitor host cell damage by redox active substances.

## 1 Data

### 1.1 Frequencies of lysine residues modified by 4-HNE

In Table 1 we list the number of lysine, histidine and cysteine residues (K, H and C, respectively) that were found conjugated with 4-HNE and compare them with the total number of each of these amino acids in the respective protein. The complete list of 4-HNE modified *Plasmodium falciparum* proteins detected after plakortin treatment and specific sites of modification are reported in [1].

There is no particular imbalance between the portion of 4-HNE-conjugated amino acids in the proteins extracted from plakortin-treated parasites. We note that *Plasmodium* proteins contain elevated % of asparagine and lysine residues [2], even more then in vertebrates, where K frequency is 2–3 times higher than that of H and C [3].

Evidently, availability of the amino acid residue for 4-HNE is a crucial factor for binding. C residues engaged in disulfide bridges are no ligands for 4-HNE, while the basic amino acid K might be more frequently exposed to the protein–solvent interface compared to H, and hence be more accessible to 4-HNE binding [4]. The data about unbalanced ratios were published for other proteins such as liver fatty acid-binding protein [5], cytochrome c [6] and alpha-synuclein [7], where 4-HNE binding site distribution between K, H, C was 4:1:1 [5]; 10:1:0 [6] and 2:1:0 [7] respectively.

### 1.2 Data for hemoglobin release from RBC under plakortin treatment

**Fig. 1:**
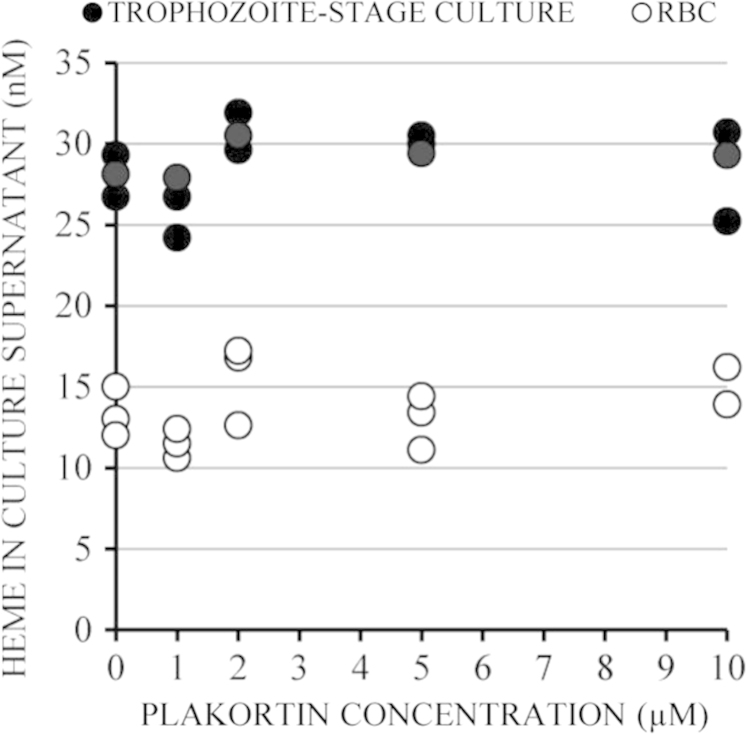
Hemoglobin release from incubated trophozoite-stage cultures and npRBC measured as heme concentration in culture supernatant. Hemoglobin release was assessed 12 h after plakortin supplementation by heme-dependent luminol-enhanced luminescence. A single point represents the mean of 3 replicates of one experiment. *N*=3 independent experiments with 3 different blood donors.

Culture of RBC infected (parasitized) with trophozoite-stage *P. falciparum* were treated with 0–10 μM of plakortin and hemoglobin release was measured in culture supernatant (Fig. 1). The concentration is indicated in nmol/l. The concentration in the whole RBC suspension was 1 mM and assessed after complete lysis of RBC in NaOH/Triton: this is the highest maximal achievable concentration of hemoglobin in the supernatant corresponding to 100% RBC lysis.

As shown in Fig. 1 the hemoglobin was not released from RBC even at 10 μM plakortin, the highest applied concentration. Very low lysis was detectable in non-parasitized RBC (npRBC) and a still very modest, although double as high value in trophozoite-parasitized mature forms of *P. falciparum*. This elevated value in trophozoites depends on the parasite biology and is plakortin independent. Plakortin has no effect on the barrier function of the host RBC membrane, irrespective whether parasitized or not.

### 1.3 Levels of reduced glutathione in plakortin-treated RBCs

**Fig. 2:**
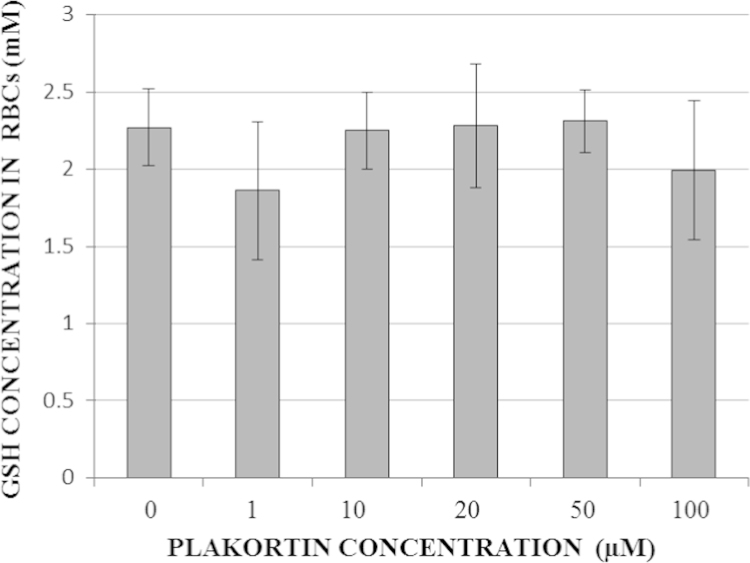
Quantification of GSH in RBC treated or not with plakortin (1–100 μM, final concentration). Columns present mean GSH±SD values from 2 replicates performed with RBC from two donors.

Reduced glutathione (GSH) was quantified in RBC kept at 5% hematocrit in PBS supplemented with 20 mM glucose and plakortin for 24 h at 37 °C. As shown in Fig. 2 the GSH level in RBC remained unchanged after plakortin supplementation in a wide concentration range from 1 to 100 μM.

## 2 Experimental design, materials and methods

Unless otherwise stated all materials were obtained from Sigma-Aldrich (St. Louis, MO, USA). Plakortin, methyl 4,8-diethyl-6-methyl-3,6-peroxy-9-dodecenoate, was obtained from the organic extract of the Caribbean sponge *Plakortis simplex* and purified by combination of column chromatography and HPLC as described [8]. The final purity of the compound was >99%.

### 2.1 in vitro cultivation of *Plasmodium falciparum* and stage-specific enrichment of parasitized RBCs

*P. falciparum* (Palo Alto strain, Mycoplasma free) parasites were kept in permanent culture as described [9], [10], [11]. RBC that were used for plakortin incubation experiments were from blood samples taken on the same day of the experiment. Cultures were incubated in a humidified CO2 cell incubator under a N_2_/CO_2_/O_2_ atmosphere of 90 vol%:5 vol%:5 vol%.

### 2.2 Plakortin treatment of parasitized RBC (pRBC) and npRBC

Pure plakortin was dissolved at 50 mM in ethanol (stock solution) and diluted to 10 mM (final concentration) with DMSO prior to use. The stock solution was kept at −20 °C until use. Synchronous cultures were supplemented with a single dose of 10 µM plakortin at ring-stage (12–16 h post-invasion) or at trophozoite-stage (26–30 h post-invasion) and kept under standard culture conditions for 3 h until mass spectrometry analysis. Enriched parasite fractions and npRBCs were treated similarly and kept under the same standard culture conditions.

### 2.3 Hemoglobin quantification

The hemoglobin concentration in the culture supernatant was assayed by heme-dependent luminol-enhanced luminescence. Luminescence was measured with a double-injector luminometer (Sirius; Berthold, Pforzheim, Germany) as described [12], [13], [14]. All assays were performed in triplicate.

### 2.4 Preparation of protein extracts from pRBC

Washed and 1700 g sedimented npRBC or pRBC were hypo-osmotically lysed in a 10-fold excess (v/v) of ice-cold lysis buffer (10 mM K_2_HPO_4_/KH_2_PO_4_ (pH 8.0)), supplemented with a protease inhibitor cocktail containing: 1 mM EDTA, 250 μM phenyl-methyl-sulfonyl fluoride (PMSF), 1 nM leupeptine, 3 μM pepstatine, the phosphatase inhibitors sodium orthovanadate and sodium fluoride each at 1 mM and 100 μM Trolox, during 5 min on ice. The ruptured membranes and organelles of parasite origin were sedimented at 16,200 g for 3 min at 4 °C and the supernatant discarded. The pellet was washed 10 times by re-suspension in fresh lysis buffer and subsequent sedimentation at 16,200 g for 1 min. pRBC preparations contained the host cell membrane as well as membranes and organelle debris from the parasite. The proteins were extracted with SDS-containing Laemmli buffer at 95 °C for 5 min. Solubilized proteins were kept at −20 °C prior to use and β-mercaptoethanol (5% v/v) was added to protein samples before loading to the SDS-PAGE. The proteins were quantified using the Bio-Rad Protein Assay (Bio-Rad, Hercules, CA, USA).

### 2.5 Identification of 4-HNE modified proteins by mass spectrometric analysis and peptide mass fingerprinting

For the identification of 4-HNE-conjugated proteins in plakortin-treated parasites, matrix assisted laser desorption ionization-time of flight (MALDI-TOF) mass spectrometric analysis was performed. Proteins extracted from enriched ring and trophozoite stage pRBCs and npRBCs cultured with or without 10 μM plakortin for 3 h were separated by SDS-PAGE. Gels were stained with colloidal Coomassie, protein bands of interest excised from the gel and in-gel trypsin digested. MS analysis was performed on a MALDI-TOF spectrometer MX; Waters, Milford, MA equipped with a delayed extraction unit, according to the tuning procedures suggested by the manufacturer, operating in reflectron mode [15], [16]. Peak lists were generated by Protein Lynx Global Server (Waters, Milford, MA). The 25 most intense masses were used for database searches against the SwissProt (Uniprot) and NCBI databases using the free search program MASCOT (http://www.matrixscience.com). The parameters used for the searches were: taxa *P. falciparum*, trypsin digestion, 1 missed cleavage, methionine oxidation and 4-HNE as variable modifications with a maximum error allowed of 100 ppm. For *P. falciparum*, proteins taken on to consideration had a Mascot score higher than 37 for SwissProt searches and higher than 67 for NCBI searches, as suggested by Mascot to be “significant”.

### 2.6 GSH assay in RBC

Assay of GSH in RBC was performed according to [17]. GSH values were given as mM (μmol/ml packed RBCs).

### 2.7 Statistical analysis

Non-parametric Mann–Whitney *U* Test was used to determine the significance of differences between the groups׳ means (PASW Statistics 18, SPSS IBM, Chicago, IL).

## Figures and Tables

**Table 1:** Lysine (K), histidine (H) and cysteine (C) frequency in the primary structure of *P. falciparum* proteins. Uniprot: Universal Protein Resource, www.uniprot.org/; NCBI GI: The National Center for Biotechnology Information GenInfo Identifier, www.ncbi.nlm.nih.gov/; PlasmoDB: the Plasmodium genome resource, http://PlasmoDB.org. Modifications at RING* stage and TROPHOZOITE^§^ stage, only.

	**4-HNE-MODIFIED PROTEIN (NAME, ALIAS; IDs)**	**4-HNE BINDING SITES (distribution between K, H, C)**	**K, H, C count in the protein sequence and the total number of amino acids**

**1**	*HSP70-1, HSP70_PLAFA	K:17	K:54
Uniprot: Q8IB24_PLAF7	H:2	H:7
NCBI GI:124512406	C:2	C:9
**PlasmoDB: PF3D7_0818900**		Tot 680 AA
**2**	*V-type proton ATPase catalytic subunit A (EC 3.6.3.14), vacuolar ATP synthase subunit a (vapA)	K:2	K:42
Uniprot: Q76NM6, VATA_PLAF7	H:0	H:6
NCBI GI:124512982	C:0	C:11
**PlasmoDB: PF3D7_1311900**		Tot 611 AA
**3**	*Enolase (EC 4.2.1.11) phosphopyruvate hydratase	K:10	K:38
Uniprot: Q8IJN7, ENO_PLAF7	H:1	H:4
NCBI GI:124802328	C:1	C:6
**PlasmoDB: PF3D7_1015900**		Tot 446 AA
**4**	*Vacuolar protein sorting-associated protein 11, putative, former RING finger protein PFE0100w	K:13	K:165
Uniprot: Q8I480-ZNRF2_PLAF7	H:0	H:31
NCBI GI:124505963	C:0	C:20
**PlasmoDB: PF3D7_0502000**		Tot 1272 AA
**5**	^**§**^**DYHC1_PLAF7; Dynein heavy chain-like protein**	K:0	K:477
Uniprot: Q 8IBG1	H:3	H:79
NCBI GI:296004907	C:0	C:69
**PlasmoDB:PF3D7_0729900**		Tot 4985 AA
**6**	^**§**^**Heat shock protein 70 (HSP70-2);** BiP; GRP78	K:4	K:63
Uniprot: Q8I2×4_PLAF7	H:6	H:6
NCBI GI:124506906	C:2	C:2
**PlasmoDB:PF3D7_0917900**		Tot 652 AA
